# Impact of Thoracic Surgery on Cardiac Morphology and Function in Small Animal Models of Heart Disease: A Cardiac MRI Study in Rats

**DOI:** 10.1371/journal.pone.0068275

**Published:** 2013-08-21

**Authors:** Peter Nordbeck, Leoni Bönhof, Karl-Heinz Hiller, Sabine Voll, Paula Arias-Loza, Lea Seidlmayer, Tatjana Williams, Yu-Xiang Ye, Daniel Gensler, Theo Pelzer, Georg Ertl, Peter M. Jakob, Wolfgang R. Bauer, Oliver Ritter

**Affiliations:** 1 Department of Internal Medicine I - Cardiology, University Hospital Würzburg, Würzburg, Germany; 2 Comprehensive Heart Failure Center, University of Würzburg, Würzburg, Germany; 3 Research Center Magnetic Resonance Bavaria, Würzburg, Würzburg, Germany; 4 Department of Experimental Physics V, University of Würzburg, Würzburg, Germany; Universityhospital Düsseldorf, Germany

## Abstract

**Background:**

Surgical procedures in small animal models of heart disease might evoke alterations in cardiac morphology and function. The aim of this study was to reveal and quantify such potential artificial early or long term effects in vivo, which might account for a significant bias in basic cardiovascular research, and, therefore, could potentially question the meaning of respective studies.

**Methods:**

Female Wistar rats (n = 6 per group) were matched for weight and assorted for sham left coronary artery ligation or control. Cardiac morphology and function was then investigated in vivo by cine magnetic resonance imaging at 7 Tesla 1 and 8 weeks after the surgical procedure. The time course of metabolic and inflammatory blood parameters was determined in addition.

**Results:**

Compared to healthy controls, rats after sham surgery showed a lower body weight both 1 week (267.5±10.6 vs. 317.0±11.3 g, n<0.05) and 8 weeks (317.0±21.1 vs. 358.7±22.4 g, n<0.05) after the intervention. Left and right ventricular morphology and function were not different in absolute measures in both groups 1 week after surgery. However, there was a confined difference in several cardiac parameters normalized to the body weight (bw), such as myocardial mass (2.19±0.30/0.83±0.13 vs. 1.85±0.22/0.70±0.07 mg left/right per g bw, p<0.05), or enddiastolic ventricular volume (1.31±0.36/1.21±0.31 vs. 1.14±0.20/1.07±0.17 µl left/right per g bw, p<0.05). Vice versa, after 8 weeks, cardiac masses, volumes, and output showed a trend for lower values in sham operated rats compared to controls in absolute measures (782.2±57.2/260.2±33.2 vs. 805.9±84.8/310.4±48.5 mg, p<0.05 for left/right ventricular mass), but not normalized to body weight. Matching these findings, blood testing revealed only minor inflammatory but prolonged metabolic changes after surgery not related to cardiac disease.

**Conclusion:**

Cardio-thoracic surgical procedures in experimental myocardial infarction cause distinct alterations upon the global integrity of the organism, which in the long term also induce circumscribed repercussions on cardiac morphology and function. This impact has to be considered when analyzing data from respective animal studies and transferring these findings to conditions in patients.

## Introduction

Surgical cardiovascular procedures, such as artificial ligation of the left anterior descendent artery (LAD) for induction of experimental myocardial infarction [1] [2], or transaortic constriction in rats and mice [3], are widely used and in some cases well established techniques in basic cardiovascular research. Until today, thousands of studies have been published investigating various specific pathophysiological questions in such models. In particular, widespread use of the mentioned experimental infarction model in rats and mice has decisively added to a deeper understanding of many aspects in myocardial ischemic wounding and healing [4] [5] [6] [7]. Meanwhile, to date the pathophysiological determinants of the surgical procedure itself - besides artificial coronary occlusion - have not been fully characterized, meaning that all studies using this model have a risk to bear a bias when trying to translate the results to the situation in incidental, inartificial myocardial infarction in animals or humans.

Even though a reference group undergoing surgical sham procedure is usually used in animal studies to possibly rule out a potential bias as good as possible - meaning that these animals undergo the same surgical procedure as those from the infarction study group besides the final step of coronary artery ligation [8] [9] [10] - there is a remaining element of uncertainty regarding the impact of the surgical manipulations on the findings in this model. This not only includes direct myocardial wounding by the ligation, or indirect surgical side effects like blood loss or inflammation, but additionally relates to damage exerted on the pericardium which is sectioned during the surgical procedure. Even though the pericardium is often surgically ligated after the procedure, it is usually not possible to fully restore its integrity. This might be a major cause of altered hemodynamics in the short term, and potentially even cause alterations in cardiac morphology in the long term. It is noteworthy that accordant surgical side-effects might not even be limited to the mentioned particular disease model of permanent myocardial infarction, as several similar and/or modified small animal models of heart disease use similar surgical approaches [11] [12]. Despite the broad usage of such animal models in basic and translational research and, therefore, high indirect implications for the clinic, we are not aware of a study comprehensively investigating the (side-)effects of the surgical procedure in experimental myocardial infarction on cardiac morphology and function in vivo.

The aim of the current study was to reveal potential side-effects of artificial thoracotomy and pericardiotomy on cardiac integrity, using cardiac magnetic resonance imaging (MRI) to characterize both left and right ventricular morphology and function in rats in vivo. In addition, the time course of metabolic and inflammatory blood markers was to be investigated. The findings of this study are intended to further validate the appropriateness of this model in cardiovascular research, potentially revealing limitations to past or future studies by experimental proof - and preferably even quantification - of possible side-effects.

## Methods

### Animal model

**Figure 1:**
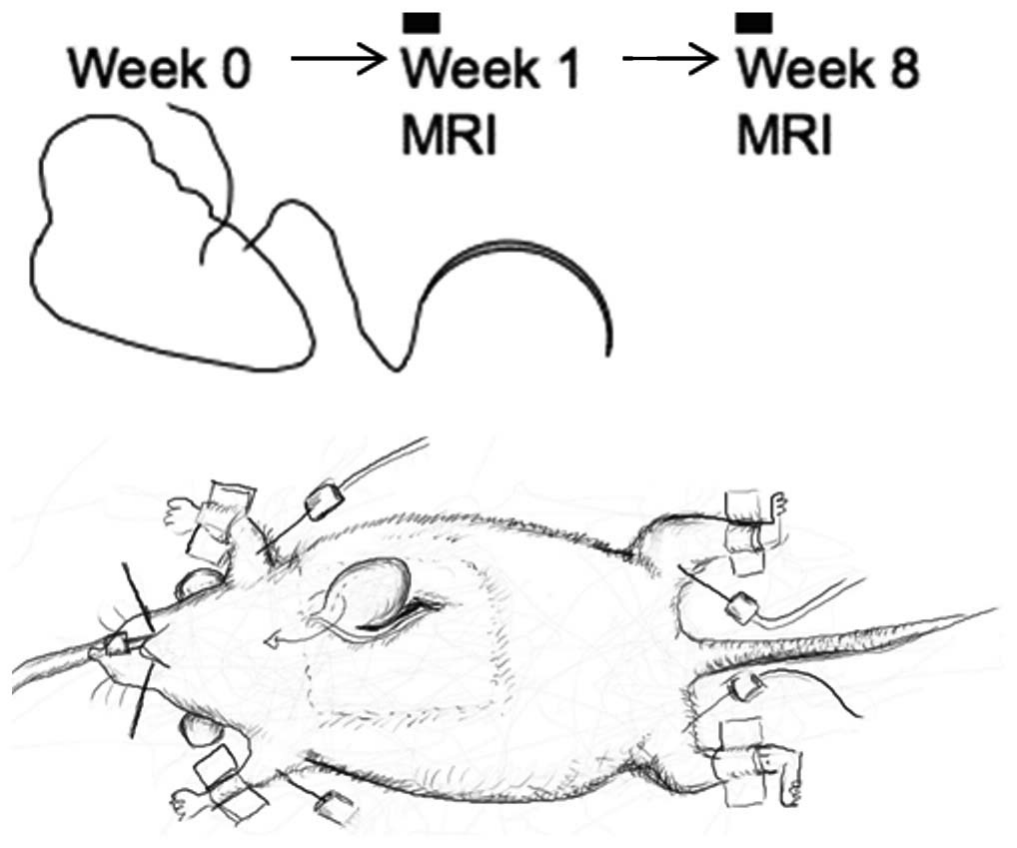
Sham surgery situs and survey arrangement. MRI was performed 1 and 8 weeks after sham surgery.

Examinations were performed in adult female Wistar rats (Charles River, Sulzfeld, Germany) with an average age of 12 weeks. The animals were divided into two groups (sham operated and untreated control rats, n = 6 per group), which were matched for body weight and followed up over time. Rats in the sham group received surgery as follows (Figure 1): Left intercostal thoracotomy was done after intubation under inhalative anesthesia (isoflurane); after exposure of the heart, pericardiotomy was performed and the heart luxated out of the thorax. The left anterior descending artery was revealed, and a suture applied around the myocardium surrounding the vessel, leaving the knot untied. The heart was then luxated back into the thorax, the pericardium closed as far as possible, and the thorax closed by suturing the several layers separately. The untreated control rats did not undergo any surgery or treatment prior to the MRI investigations.

Magnetic resonance investigations started one week after the sham operation for detection of potential early side effects, and were repeated eight weeks after sham surgery to reveal potential late appearing side effects. The untreated rats were used as controls at equivalent time points.

All animal work was conducted according to the relevant national and international guidelines. The experimental protocol was approved by the local ethics committee of the University of Wuerzburg and the governmental animal care and use committee (Regierung von Unterfranken).

### Magnetic resonance imaging

Experiments were performed on a Bruker Biospec 70/21 using a whole body birdcage coil (Bruker Biospin, Ettlingen, Germany) for transmission and a circular polarized surface coil as receiver on intubated animals under inhalation anesthesia with isoflurane.

**Figure 2:**
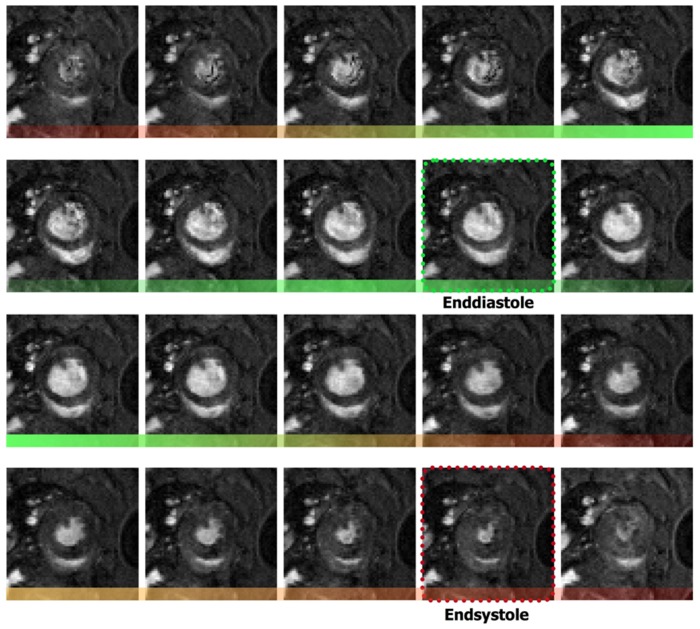
Representative full cycle of a cardiac short-axis cine-MRI with 20 frames.

An ECG-triggered fast gradient echo (FLASH) cine sequence was used [13]. Flip angle was 30–40°, echo time was 1.1 ms, and repetition time 3.2 ms. We used a field of view of 50 mm×50 mm and an image matrix of 256×256. 16–37 cine frames per heart cycle were obtained to temporally cover the whole cardiac cycle. 16–18 contiguous ventricular short axis slices of 1 mm thickness were acquired to spatially acquire the entire heart (Figure 2). The total acquisition time for one cine sequence was 40–50 s depending on heart rate. Total heart scan time was approximately 15 min for each animal.

Both ventricles were equally investigated; we chose standard short axis ventricular multi slice image stacks for quantification of cardiac morphological and functional parameters as most MRI studies are left ventricular optimized.

### Blood analyses

In addition to the MRI investigations, serial blood analyses were performed in control and sham operated rats, investigating the time course of several metabolic and inflammatory blood markers. To this end, blood was extracted via the tail vein from the animals at baseline and 1, 4, 7, and 14 days after sham surgery. Quantitative analyses were then performed using enzyme-linked immunosorbent assay (ELISA) according to the manufacturer's protocol. Metabolic testing included determination of the following blood serum parameters: glucose, urea, triglycerides, and leptin. Inflammatory testing included determination of the following blood serum parameters: c-reactive protein (CRP), tumor necrosis factor alpha (TNF), and interleukin 2 (IL-2).

### Histology

After completion of the experiments, the rats were euthanized. The hearts were excised, sliced, fixated, and stained using Picrosirius Red. Histology of the whole hearts was then performed to rule out inadvertent small myocardial infarction, which might have escaped detection in MRI and could potentially tamper the morphological and functional findings.

### Data analysis

**Figure 3:**
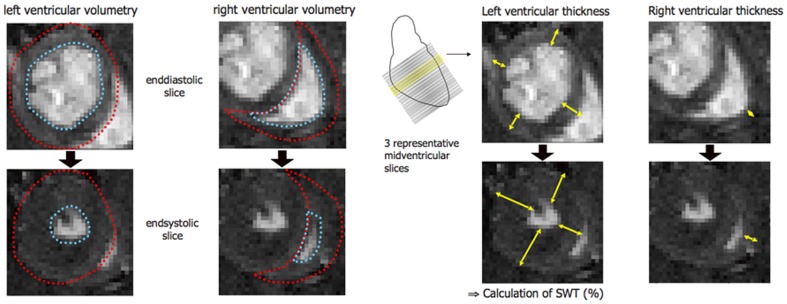
Illustration of left and right ventricular volumetry and determination of wall thickness and systolic function.

Data analysis was done using an operator-interactive threshold technique (IDL 5.2 Software). Visual analysis was used to determine the endsystolic and enddiastolic frame for every slice. Ventricular slice areas were then manually encircled in the respective frames, and ventricular volumes per slice then calculated by multiplication of determined area and slice thickness (Figure 3). Finally, total volumes were determined as the sum of all slice volumes. Left and right ventricular masses were calculated as myocardial volume multiplied by the myocardial specific gravity (1.05 g/cm^3^). For comparison of sham operated vs. untreated control animals the following cardiac parameters were included in the analysis: Left and right ventricular myocardial masses (LVM and RVM), enddiastolic and endsystolic wall thickness (EDWT and ESWT), enddiastolic and endsystolic volumes (EDV and ESV), absolute and percental systolic wall thickening (SWT, SWT%), stroke volume (SV), ejection fraction (EF), and cardiac output (CO).

Stroke volume was calculated as the difference between end-diastolic and end-systolic volumes after summing all slice volumes. For calculation of cardiac output, stroke volume was multiplied by heart rate. Ejection fraction was calculated by division of stroke volume and enddiastolic volume. Comparison of endsystolic and enddiastolic ventricular masses was used as a parameter to validate the preciseness of the measurements and postprocessing. Wall thickness and systolic wall thickening were examined as follows: Three representative midventricular slices were chosen and left and right ventricular wall thickness then measured manually in enddiastolic and endsystolic frames. The right ventricular wall thickness and thickening was measured only in the lateral part of the wall, while the left ventricular myocardial thickness was measured in an anterior, lateral, posterior, and septal segment for each slice. Mean wall thickening (SWT in total and %) was calculated for each slice, then overall mean wall thickening was calculated over the three slices. In addition to the SWT determination, the cine loops were visually analyzed to detect not only wall thinning and akinesia, but also potential dyskinesia.

Data analysis was performed by two persons and identical equipment throughout the whole study to reduce intraobserver and interobserver variability. To follow convention, papillary muscles were not included in myocardial masses.

### Statistical analysis

SPSS Statistics 19 (IBM, Ehningen, Germany) was used for statistical analyses. All data are given in mean ± standard deviation (SD). In calculated data, error was computed by Gaussian propagation of uncertainty and taken as the random error if it was larger than standard deviation. A two-tailed students t-test for paired group comparison was applied. P<0.05 was considered statistically significant.

## Results

Six animals were included in each group. Mean body weight at baseline was 296±7.4 (sham) and 301±6.3 g (control). Sham surgery was performed successfully in all animals for the sham group without apparent acute complications.

**Figure 4:**
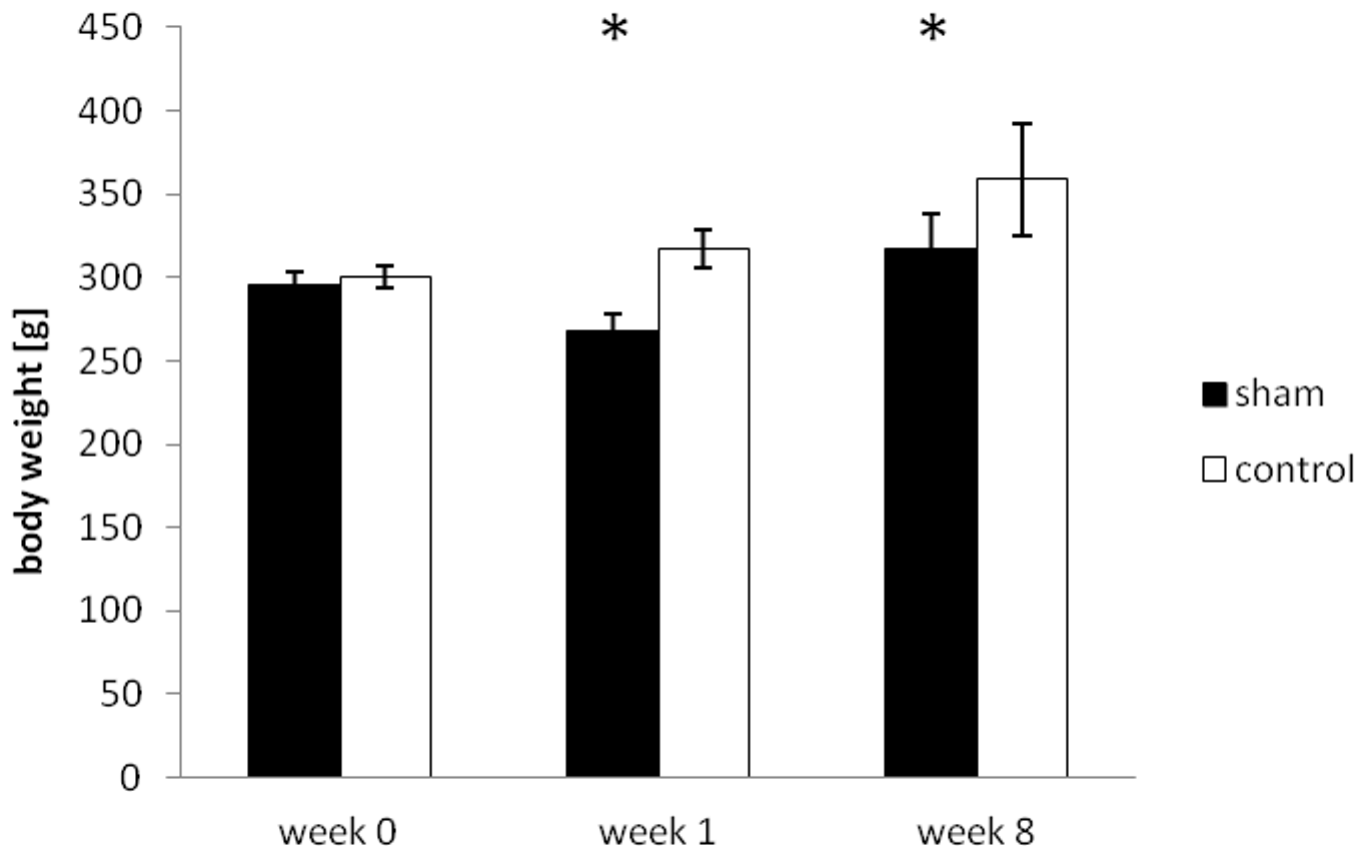
Body weight at baseline, 1 week and 8 weeks after sham surgery in both groups (mean ± SD). * indicates significant differences (p<0.05) between the respective groups.

**Figure 5:**
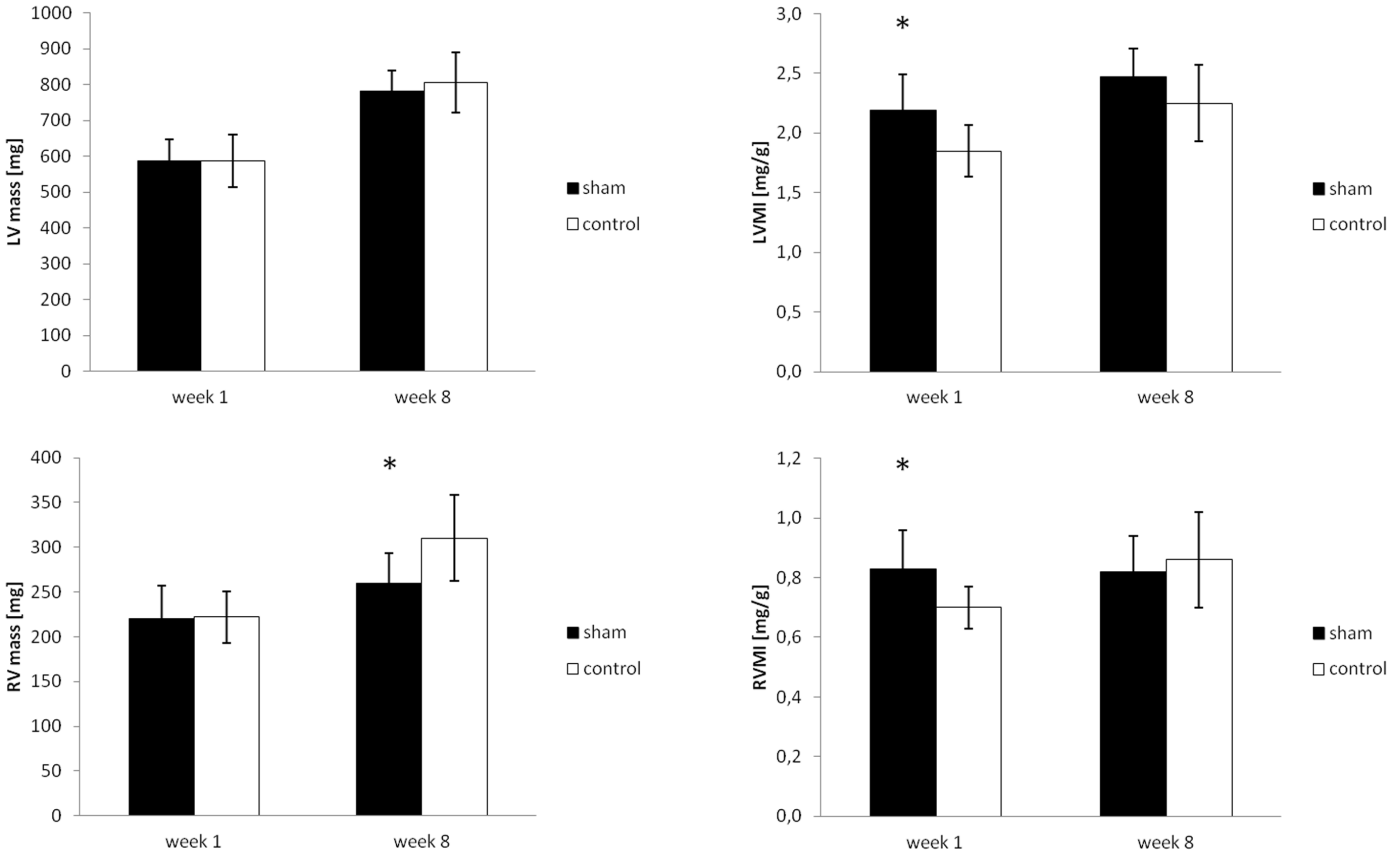
Left and right ventricular myocardial mass and myocardial mass index 1 week and 8 weeks after sham surgery (mean ± SD). * indicates significant differences (p<0.05) between the respective groups.

**Figure 6:**
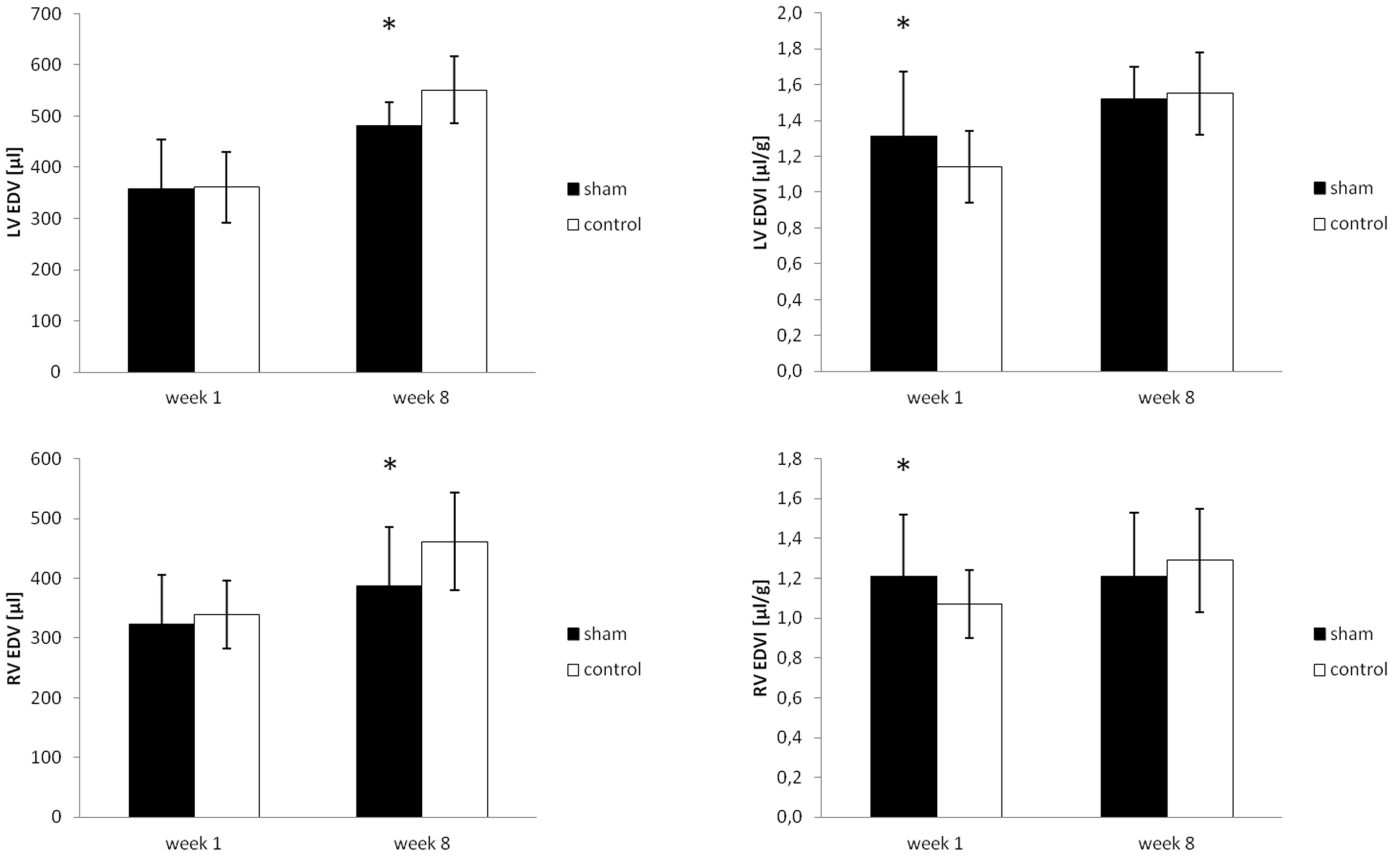
Left and right ventricular enddiastolic volume and enddiastolic volume index normalized to body weight 1 week and 8 weeks after sham surgery (mean ± SD). * indicates significant differences (p<0.05) between the respective groups.

**Figure 7:**
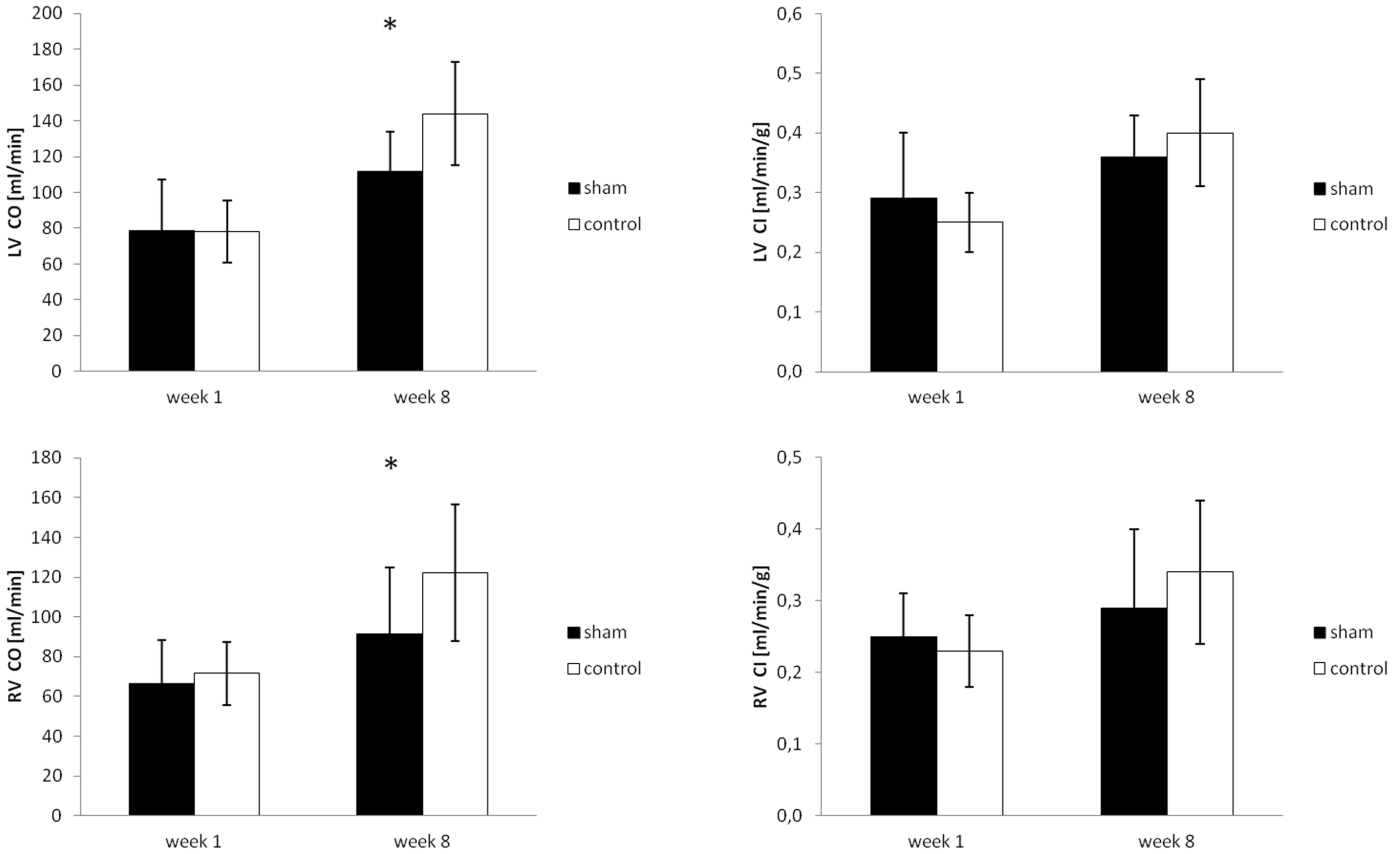
Left and right ventricular cardiac output and cardiac index normalized to body weight 1 week and 8 weeks after sham surgery (mean ± SD). * indicates significant differences (p<0.05) between the respective groups.

One week after sham surgery, rats in the sham group showed a marked decrease in body weight (268±10.6 vs. 317±11.3 g, p<0.05) accounting for a weight loss of −28.2±7.4 g, compared to an increase in body weight of +16.3±7.9 g in the control group (Figure 4). Heart rate during MRI was similar in both groups (279±27 vs. 283±26 bpm, p = n.s.). No difference in left or right ventricular wall thickness (1.95±0.5/1.0±0.28 for sham vs. 1.90±0.14/0.9±0.07 mm, p = n.s., in enddiastole for LV/RV and sham vs. control), or myocardial masses (587.0±60.7/220.6±36.6 vs. 587.8±73.5/222.1±29.0 mg, p = n.s., Figure 5) could be found. Equally, enddiastolic ventricular volumes (358.2±96.6/323.1±82.0 vs. 361.0±69.2/339.1±56.9 µl, p = n.s., Figure 6), endsystolic ventricular volumes (79.8±22.7/85.9±17.9 vs. 85.6±20.3/81.0±16.5 µl, p = n.s.), stroke volumes (278.4±99.2/237.1±68.3 vs. 275.4±50.8/252.1±46.6 µl, p = n.s.), ejection fraction (78±4/73±4 vs. 76±2/73±3%, p = n.s.), and cardiac output (78.6±28.7/66.6±21.8 vs. 78.2±17.5/71.6±15.7 ml/min, p = n.s., Figure 7) were not different in both groups. Normalized to the body weight, ventricular masses and enddiastolic volumes were significantly higher in the sham operated compared to the control group (2.19±0.30/0.83±0.13 vs. 1.85±0.22/0.70±0.07 mg myocardium per g body weight, p<0.05, and 1.31±0.36/1.21±0.31 vs. 1.14±0.20/1.07±0.17 µl ventricular volume per g body weight, p<0.05) 1 week after surgery. Meanwhile, cardiac output normalized to the body weight was not statistically different in both groups (0.29±0.11/0.25±0.06 vs. 0.25±0.05/0.23±0.05 ml per min and g body weight, p = n.s.).

Eight weeks after sham surgery, rats in both groups showed a (further) increase in body weight compared to week 1 with a trend for a higher increase in the sham animals (+49.5±21.7 vs. +41.7±26.4 g, p = n.s.), even though the resulting mean body weight still remained significantly lower in the sham operated group compared to the control group (317±21.1 vs. 259±33.4 g, p<0.05) (Figure 4). Compared to rats in the sham operated group, animals in the control group showed a slightly higher heart rate (306±30 vs. 355±29 bpm, p<0.05). At this time point, absolute values of left and right ventricular masses showed a tendency for lower values in sham operated vs. control animals (782.2±57.2/260.2±33.2 vs. 805.9±84.8/310.4±48.5 mg, p<0.05 for RV mass), while the respective indices, normalized to body weight, were not different (2.47±0.24/0.82±0.12 vs. 2.25±0.32/0.86±0.16 mg myocardium per g body weight, p = n.s.). Equally, compared to sham operated animals, enddiastolic ventricular volumes were slightly higher in the control group (480.9±46.7/386.5±98.7 vs. 551.1±65.3/461.7±81.2 µl, p<0.05), while no significant difference in the normalized values could be seen (1.52±0.18/1.21±0.32 vs. 1.55±0.23/1.29±0.26 µl ventricular volume per g body weight, p = n.s.). Cardiac output normalized to the body weight was also not statistically different in both groups, even though there was a trend for higher respective values in the control group compared to the shams (0.36±0.07/0.29±0.11 vs. 0.40±0.09/0.34±0.10 ml per min and g body weight, p = n.s.). A complete overview of the MRI results is given in Table 1 for the left ventricular and Table 2 for the right ventricular data.

**Table 1:** Results overview, left ventricle.

LV	Sham 1 week 1,	± SD n = 6	Control 1 week 1,	± SD n = 6	Sham 8 week 8,	± SD n = 6	Control 8 week 8,	± SD n = 6
Body mass (g)	**267.5** *	10.6	**317.0** *	11.3	**317.0** *	21.1	**358.7** *	33.4
HR (1/min)	**279**	27	**283**	26	**306** *	30	**355** *	29
LVM (mg)	**587.0**	60.7	**587.8**	73.5	**782.2**	57.2	**805.9**	84.8
EDWT (mm)	**1.95**	0.54	**1.90**	0.14	**1.82**	0.06	**1.92**	0.17
ESWT (mm)	**3.26**	0.62	**3.35**	0.22	**3.35**	0.22	**3.40**	0.32
EDV (µl)	**358.2**	96.6	**361.0**	69.2	**480.9** *	46.7	**551.1** *	65.3
ESV (µl)	**79.8**	22.7	**85.6**	20.3	**116.3** *	42.9	**143.8** *	31.7
SWT (mm)	**1.31**	0.21	**1.44**	0.12	**1.53**	0.19	**1.48**	0.18
SWT (%)	**71**	19	**76**	6	**84**	9	**77**	7
SV (µl)	**278.4**	99.2	**275.4**	50.8	**364.7**	63.4	**407.3**	72.6
CO (ml/min)	**78.6**	28.7	**78.2**	17.5	**111.7** *	22.2	**143.9** *	28.7
EF (%)	**78**	4	**76**	2	**76**	3	**74**	3
LVM/EDV (g/ml)	**1.75**	0.49	**1.65**	0.15	**1.63**	0.41	**1.47**	0.44
LVMI (mg/g)	**2.19** *	0.30	**1.85** *	0.22	**2.47**	0.24	**2.25**	0.32
EDVI (µl/g)	**1.31** *	0.36	**1.14** *	0.20	**1.52**	0.18	**1.55**	0.23
ESVI (µl/g)	**0.31**	0.09	**0.27**	0.06	**0.36**	0.14	**0.40**	0.90
SVI (µl/g)	**1.04**	0.37	**0.87**	0.15	**1.16**	0.21	**1.15**	0.23
CI (ml/min/g)	**0.29**	0.11	**0.25**	0.05	**0.36**	0.07	**0.40**	0.09

All results are given as mean±SD. LV: left ventricle, HR: heart rate, LVM: left ventricular myocardial mass, EDWT: enddiastolic wall thickness, ESWT: endsystolic wall thickness, EDV: enddiastolic volume, ESV: endsystolic volume, SWT: systolic wall thickening, SV: stroke volume, CO: cardiac output, EF: ejection fraction, and (C)I: (cardiac) index (per gram body weight).

* indicates significant differences (p<0.05) between the respective groups.

**Table 2:** Results overview, right ventricle.

RV	Sham 1 week 1,	± SD n = 6	Control 1 week 1,	± SD n = 6	Sham 8 week 8,	± SD n = 6	Control 8 week 8,	± SD n = 6
Body mass (g)	**267.5** *	10,6	**317.0** *	11,3	**317.0** *	21.1	**358.7** *	33.4
HR (1/min)	**279**	27	**283**	26	**306** *	30	**355** *	29
RVM (mg)	**220.6**	36.6	**222.1**	29.0	**260.2** *	33.2	**310.4** *	48.5
EDWT (mm)	**1.00**	0.28	**0.90**	0.07	**1.00**	0.04	**0.94**	0.06
ESWT (mm)	**1.68**	0.52	**1.55**	0.16	**1.57**	0.17	**1.48**	0.19
EDV (µl)	**323.1**	82.0	**339.1**	56.9	**386.5** *	98.7	**461.7** *	81.2
ESV (µl)	**85.9**	17.9	**81.0**	16.5	**87.9** *	36.6	**115.8** *	44.5
SWT (mm)	**0.68**	0.29	**0.66**	0.12	**0.57**	0.14	**0.54**	0.16
SWT (%)	**68**	20	**73**	12	**56**	12	**58**	17
SV (µl)	**237.1**	68.3	**252.1**	46.6	**298.6**	105.0	**356.8**	92.6
CO (ml/min)	**66.6**	21.8	**71.6**	15.7	**91.6** *	33.4	**122.0** *	34.4
EF (%)	**73**	4	**73**	3	**77**	8	**75**	7
RVM/EDV (g/ml)	**0.70**	0.20	**0.67**	0.07	**0.70**	0.42	**0.68**	0.36
RVMI (mg/g)	**0.83** *	0.13	**0.70** *	0.07	**0.82**	0.12	**0.86**	0.16
EDVI (µl/g)	**1.21** *	0.31	**1.07** *	0.17	**1.21**	0.32	**1.29**	0.26
ESVI (µl/g)	**0.32**	0.14	**0.26**	0.05	**0.27**	0.12	**0.32**	0.13
SVI (µl/g)	**0.89**	0.24	**0.81**	0.13	**0.94**	0.34	**0.97**	0.27
CI (ml/min/g)	**0.25**	0.06	**0.23**	0.05	**0.29**	0.11	**0.34**	0.10

All results are given as mean±SD. RV: right ventricle, HR: heart rate, RVM: right ventricular myocardial mass, EDWT: enddiastolic wall thickness, ESWT: endsystolic wall thickness, EDV: enddiastolic volume, ESV: endsystolic volume, SWT: systolic wall thickening, SV: stroke volume, CO: cardiac output, EF: ejection fraction, and (C)I: (cardiac) index (per gram body weight).

* indicates significant differences (p<0.05) between the respective groups.

Histology after completion of the MR measurements showed no significant areas of myocardial infarction (defined as scar areas covering >10% of the left ventricular myocardium) in any of the sham operated animals.

**Figure 8:**
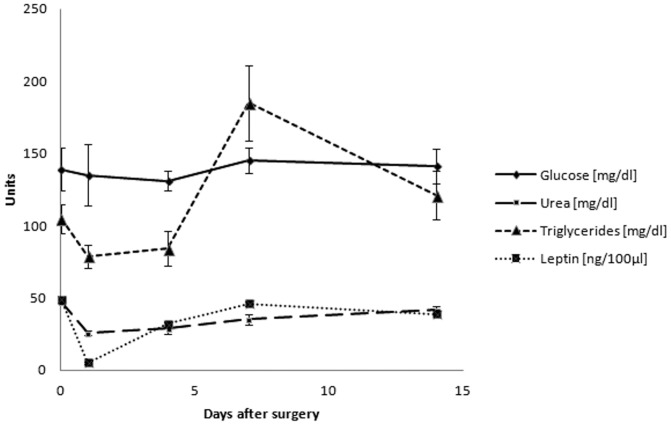
Time course of various metabolic blood parameters (glucose, urea, triglycerides, and leptin) after sham surgery.

Blood analyses using ELISA did not show any significant alterations in CRP, TNF, or IL-2 in the short or long term after sham surgery. Analyses of metabolic blood parameters showed normal glucose, but a significant decrease in urea and triglycerides 1 and 4 days after sham surgery, recovering to normal levels 7 days after surgery (Figure 8). Along with these findings, blood leptin levels were also diminished in the short, but not long term (Figure 8).

## Discussion

Small animal models of cardiac diseases have been extensively used in basic cardiovascular research to investigate the pathophysiology of various medical conditions and subsequent potential therapeutic approaches. Artificial myocardial infarction by surgical ligation of the left descending coronary artery is a particularly popular disease model [1] [2], with thousands of studies performed until then using this specific method or similar variants. However, the use of such artificial disease models is afflicted with a substantial risk of biases. First, certain preconditions and requirements, which are often obligatory for natural development of certain diseases, might be missing in such artificial disease models. This includes certain genetic conditions, behavioral aspects such as nicotine consumption or exercise frequency, but also underlying or co-morbidities specifically accompanying a naturally developed disease. Second, artificial procedures undertaken to provoke a pathological condition strived for might cause specific side-effects that might directly influence the appearance or development of a disease. The existence of one or more of these biases might question the results and meaning of such studies, which is why a comprehensive understanding of such potential side effects is desirable.

The current study aimed at a deeper understanding of the artificially induced pathophysiological aspects and/or side-effects in the above mentioned small animal model of myocardial infarction induced by surgical ligation of a coronary artery excluding the specific effects of coronary artery occlusion itself. Execution of this particular procedure not only means a disturbance of global integrity of the organism e.g. by blood loss or development of a subsequent inflammatory response due to the surgery. Because the surgical procedure includes a direct, mechanical intervention at the (epi-)myocardium, the heart's integrity might be distinctly disturbed, e.g. by intramyocardial bleeding or the secondary effects of pericardiotomy/pericardectomy. It might be reasonable to suggest that the latter might substantially alter cardiac hemodynamics particularly regarding filling of the right ventricle. In order to investigate and quantify such potential effects we used high field MRI to characterize development of cardiac morphology and function in vivo over time after the surgical procedure.

MRI offers exceptional options for exact characterization not only of the left, but also the right ventricle, and therefore is suitable to detect even small alterations which might remain undetected by other imaging modalities like e.g. echocardiography [14]. Mainly due to its non-invasiveness and excellent soft tissue contrast along with high spatial and temporal resolution, cardiac MRI has been established as the gold standard to exactly measure cardiac functional and morphologic parameters over the last years. Therefore, it is not surprising that an increasing number of myocardial infarction-related studies in small animal models [15] [16] [17], including several studies of our own research group [4] [8], have used MRI for in vivo evaluation of myocardial morphology and function in the past and present.

Based on the results of the current study, a main effect of the sham operation was an acute slight decrease of the animals' body weight, presumably as a direct consequence of the surgical procedure. Following the acute phase, weight gain in these rats was apparently not further diminished, but these rats rather caught up part of this decrease in body weight loss compared to the control animals. However, at week 8, there was still a significant remaining difference comparing the total body weight in both groups. According to the current findings from blood analyses, it is unlikely that inflammatory processes account for significant alterations in the short or long term: quantitative ELISA showed only minor, non-significant alterations in CRP, TNF, or IL-2 after sham surgery. Metabolic blood parameters, however, confirmed distinct metabolic alterations due to sham surgery, including a significant drop in urea and triglycerides from 1 till 4 days post-surgery. In addition, serum leptin levels - which are suggested to play a major role in regulation of food intake, but have also been shown to be reversely influenced by behavioral and metabolic changes in food intake [18] - were also found to be diminished in the acute phase after sham surgery. These findings suggest a major impact of metabolic alterations on body integrity after sham surgery, which should be investigated further in the future.

It can also be concluded from the results of the study that the acute, direct side-effects of the surgical procedure on cardiac morphology and function are negligible one week after the intervention. There was no acute substantial alteration in myocardial mass, wall thickness, ventricular volumes, or function in comparison to the control animals, neither for the left nor right ventricle. In addition to the global functional cardiac parameters, visual analysis of the cine cycles also showed no regional impairment of cardiac function in the sham operated animals. It can be concluded from the results of the study, that the integrity of the pericardium has only a small effect on both left and right ventricular morphology and function. Right ventricular function was impaired neither at week 1 nor at week 8 after surgery. The only apparent effect seen in the rats after the intervention was a tendency for higher right ventricular wall thickness at least in some of the animals, even though not statistically significant comparing the entire groups. This increase in the myocardial wall thickness might in fact be an incorrect labeling, as even using MRI it is not always possible to discriminate the right ventricular myocardium from the pericardium. Therefore, rather than effects on the myocardium itself, pericardial swelling or hemorrhage might be primarily responsible for this (limited) effect seen in the MRI, which was less pronounced 8 weeks after surgery.

In contrast to the acute phase after sham surgery, which led to no significant alterations in cardiac morphology or function in absolute measures, but some measureable differences of cardiac parameters in relation to the respective body weight, there were some differences in cardiac parameters between both groups in absolute measures at week 8 after sham surgery, but no longer normalized to the animal body weight. This implicates physiological secondary long term customization of the cardiac morphology and function on the aroused alterations in body weight, rather than suggesting a pathological cause. A marked difference between both groups in the long term was a substantially higher heart rate in the control animals compared to the sham animals, and also compared to both groups at the early time point. From the results of the study, the reasons for this increase in heart rate in the respective group compared to all other groups are unclear. It could be speculated though, that the main reason for this increase might simply be the higher body weight, which might imply less cooling of the animal under anesthesia and subsequently lead to a higher body temperature with higher heart rate during the MR investigation. The accompanying trend for an increased cardiac index in these animals might be explained equivalently as a direct result of the higher heart rate, since stroke volume index normalized to the body weight was identical in both groups at week 8.

## Conclusion

Surgical procedures undertaken to evoke artificial myocardial infarction by ligation of a branch of the coronary arteries induce a transient general impairment of the body integrity, apart from the local effects on the connective tissue mainly apparent by a slight decrease in body weight in the short term. Despite pericardiotomy and circumscribed intramural wounding through the procedure for coronary ligation, there are only minimal acute effects on left and right ventricular morphology and function. Correlation of many cardiac parameters with body weight can, however, show alterations compared to healthy controls. In the long term, the transient impairment in body weight is partly regained, with cardiac morphology gradually adapting according to the development of the organism similar to healthy individuals. These findings should be taken into account when evaluating results from basic cardiovascular research in small animal models of cardiac disease, particularly if the results from studies including thoracic surgery are directly compared to those from studies that do not include thoracic surgery.
